# Effect of mobile learning on academic achievement and attitude of Sudanese dental students: a preliminary study

**DOI:** 10.1186/s12909-021-02509-x

**Published:** 2021-02-22

**Authors:** Nissreen Nugud Mergany, Alam-Elhuda Dafalla, Elhadi Awooda

**Affiliations:** 1 Pediatric Dentistry Department/ Faculty of Dentistry, International University of Africa, Khartoum, Sudan; 2 Department of Community Medicine & Public Health, Al-Neelain University, Khartoum, Sudan; 3 Educational Development and Research Center, University of Gizera, Wad Madani, Sudan

**Keywords:** Academic achievement, Dental students’ attitude, Educational applications, Mobile learning

## Abstract

**Background:**

Despite the great development of smart phone programs and applications, and the wide-spread of these devices among students, their use for the educational purposes remains weak. The study aimed to investigate to what extent utilizing mobile learning as an adjunct to classic classroom lectures affect students’ academic achievement and, to assess their attitude toward using mobile application as an instructional method in dental education.

**Methods:**

A quasi-experimental study was conducted among undergraduate dental students from two Sudanese universities. A total of 67 students who voluntarily agreed to participate were randomly allocated into a control group of 33 and an intervention group of 34 students. Initially, the two groups undertook a pre-test to ensure the standardization of a scale regarding their existing academic knowledge of dental surgery forceps used for tooth extraction. Then the intervention group was provided with a mobile application (Dental Surgical Forceps application version 2.1.0.0), and 3 weeks later a post-test was given for both groups**.** The attitude of the students toward the effectiveness of mobile learning was as assessed by five-point Likert scale questionnaire. For comparison of the numerical parametric data, a T. test was used, while for non-parametric categorical data a Chi-Squire test was used, with level of statistical significant difference set at *P*-value of ≤0.05.

**Results:**

The response rate was 91% for the intervention group (31 out of 34 students completed the study), and 78% for the control group (26 out of 33 students completed the study). Statistical significant difference was observed between the pretest and post-test mean scores of the intervention group (*P* < 0.005), while the differences were not significant among the control group (*P* > 0.05). Regarding the attitude of the dental students, the mean scores of the sample indicate that the vast majority of the participants (93.5%) showed positive attitude regarding the effectiveness of mobile learning.

**Conclusions:**

There is a marked difference in the students’ scores regarding their knowledge of dental surgical forceps. The students showed positive attitude toward using the mobile application.

**Supplementary Information:**

The online version contains supplementary material available at 10.1186/s12909-021-02509-x.

## Background

Through the last two decades, researchers have advocated for many definitions and descriptions of mobile learning; they have coined terms like learning virtually, learners’ mobility, anywhere and anytime, and via mobile devices [1]. But in short it can be defined as: “Mobile learning involves the use of mobile technology, either alone or in combination with other information and communication technology (ICT), to enable learning anytime and anywhere” [2].

Statistics have shown that the highest numbers of mobile users are in the age group of 18–34 years [3]. If we take into account all features of smart phones and tablets, which millions of students already own, this represents a powerful tool for learning that can effectively influence education. The status of m-learning in Sudanese universities is not clear, but many factors impeding adoption and implementation of m-learning have defined. The factors can summarized as; the lack of strategic plan, the absence of m-learning policy, financial resources and infrastructure, digital skills, and internet availability [4].

One of the skills undergraduates dental students are required to be competent in is tooth extraction [5] it represents about one-third of a dental practice [6]. Although there are variations in the content and delivery of oral surgery teaching programs between individual dental schools, they are usually divided into pre-clinical and clinical phases. Lectures are the most widely used method in the pre-clinical oral surgery modules, but they are not the most effective method from students’ perspective [3] _._ If we take into account the insufficient numbers of instructors in some dental schools [7]_,_ we can appreciate that 6–60% of students reported that they were unsatisfied regarding their knowledge of forceps and elevators in a study performed by the Academic Centre of Dentistry Amsterdam, which investigated students’ opinion about theoretical and clinical tutoring in tooth extraction at different European dental schools [5].

The shortage in the number of clinical instructor versus a large number of students in the Sudanese dental schools may not give the students enough opportunity to observe and follow up the instructor’s explanation of dental instruments. Moreover, knowledge and familiarity with the available in all teaching institutions. So use of dental surgical forceps application can meet this dental surgery forceps require exposure to many cases of tooth extraction; this may not be need.

This interventional study is a trial to provide evidence-based data by exploring the following research questions: 1) To what extent utilizing mobile applications as an adjunct to classic classroom lectures affect students’ academic achievement? How using mobile learning applications affect the attitude of students?

## Methods

### Overview

The study was conducted from May to July 2018 at the Faculty of Dentistry, International University of Africa, and the Faculty of Dentistry, The National University. The dental program (Bachelor of Dental Medicine and Surgery) (B.D.M.S) in both faculties is 5 years (10 semesters) divided into three phases: The first 2 years (Semesters 1–4) comprise studying of the basic sciences, and usually be at the main campus of the University with 1 or 2 days off campus in visits to relevant institutions and training facilities. The second phase is called pre clinical (semester 5–6), where the students start to perform the clinical procedures on artificial teeth (phantom-head lab). The last 2 years (Semesters 7–10) is the clinical phase, based at clinical training sites, mainly at the campus clinics. We have selected students from the eighth and ninth semester because they were at the same level of training, students at the beginning of the seventh semester may not have started yet to study the surgical forceps, while the students at the end of the tenth semester have completed their practical training and have enough knowledge on the topic.

A quasi-experimental design (Pre-test/post-test nonequivalent group approach) was implemented to evaluate the effect of smartphone educational application on dental students’ attitudes and theoretical knowledge.

### Participants

The convenience sampling technique was used. Sixty-seven undergraduate dental students at semester eight and semester nine agreed to participate in the study, 31 students from the International University of Africa and, 36 students from the National University**.**

### Inclusion criteria

- Undergraduate dental students enrolled in the eighth and ninth semester
- Students who have smart phone or tablet
- Students who agree to participate in the study

### Exclusion criteria

- Undergraduate dental students not enrolled in the eighth and ninth semester
- Students who don’t have smart phone or tablet
- Students who disagree to participate in the study

### Materials

1. Software application for Android (Dental Surgical Forceps Application version 2.1.0.0)
  The app. Consist of six screens, containing a gallery of text, photo-based materials that describe the dental surgical forceps and their uses (Additional File 1).

2) Academic assessment test (pre-test and post-test) to determine whether the use of a dental surgical forceps mobile application affected students’ academic achievement. The test consisted of 10 photos of surgical dental forceps, and the students were asked to identify each instrument and the purpose of its use (See Supplementary Table 1, Additional File 2).
3) A five-point Likert-Scale questionnaire—with strongly agree, agree, undecided, disagree, and strongly disagree—was used to study the students’ attitudes regarding the effectiveness of m- learning (See Supplementary Table 1, Additional File 3). The questionnaire was adopted from previous similar study [8, 9]. The reliability analysis of the questionnaire was assessed by the internal consistency that represented by Cronbach’s alpha, while the validity (construct validity) was estimated by factor analysis that divided the eight questionnaire statements into two components; statements 1–5, and statements 6–8. Alpha value of the first component is 0.8419 and the alpha value of the second component is 0.6079 [8].

### Procedure

**Fig. 1:**
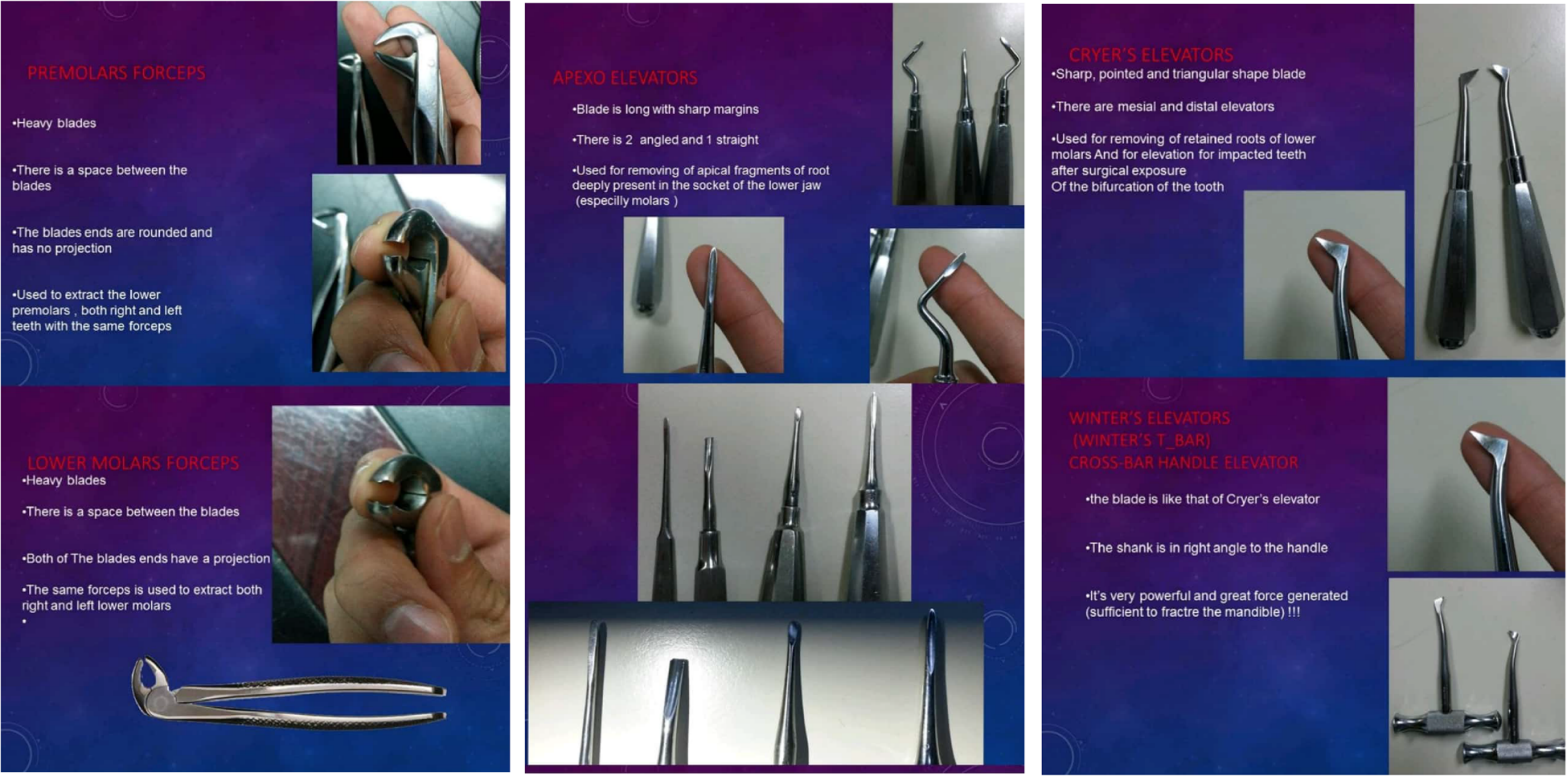
Screenshots of dental surgical forceps app. Defining different forceps and elevators

**Fig. 2:**
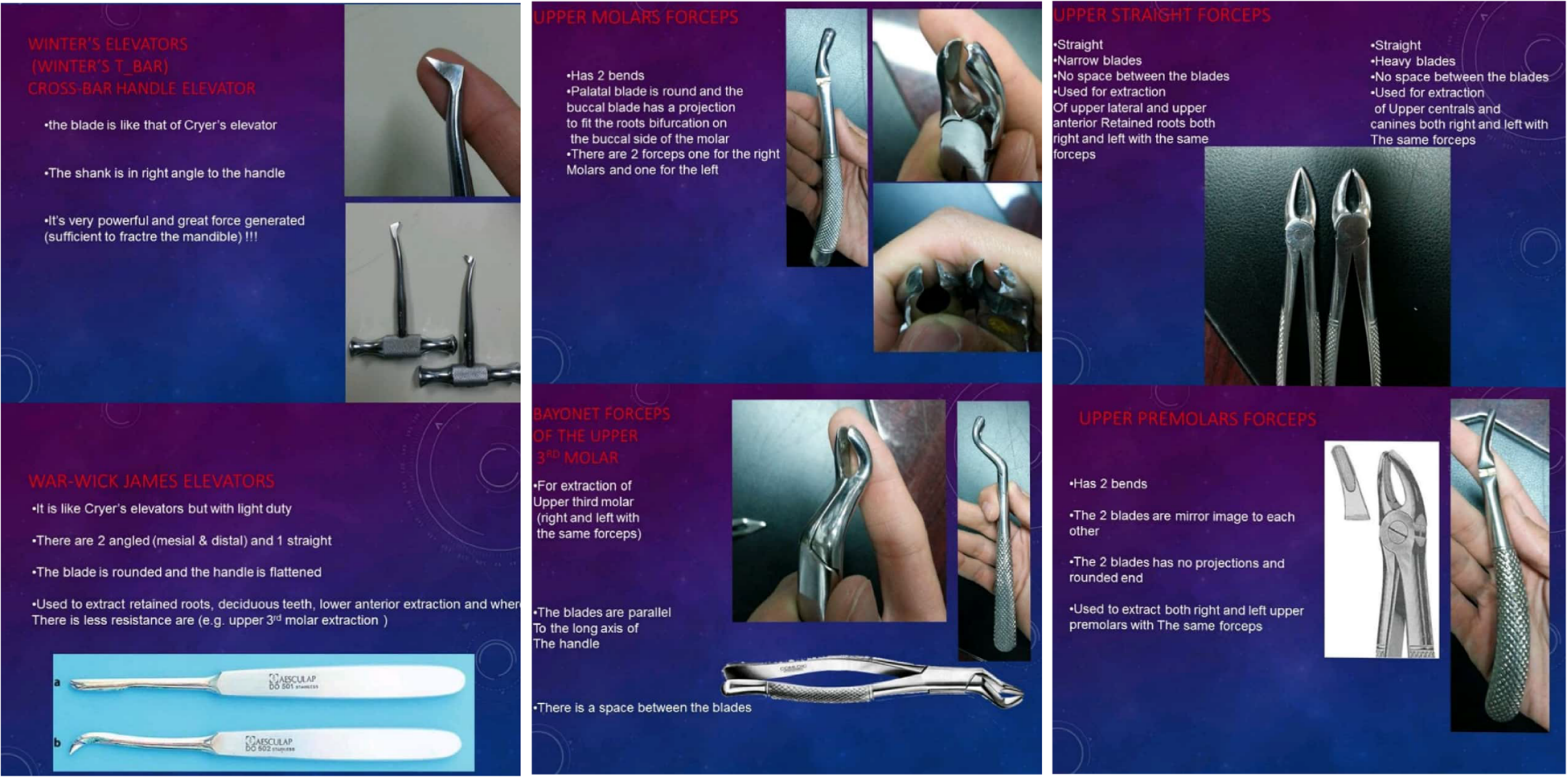
Screenshots of dental surgical forceps app. Defining different forceps and elevators

Initially, the two groups undertook a test (pre-test) to make sure of the standardization of a scale regarding their existing academic knowledge. Then the intervention group was provided by the App that containing a gallery of the dental surgical forceps (Figs. 1-2) while the control group received no intervention, and 3 weeks later a post-test was applied for both groups**.** The mean score of the intervention group was compared with the control group to find if there was a significant difference**.**

### Data analysis

The data were entered in an Excel spreadsheet and then analyzed using Microsoft Excel 2016 and Statistical Package for the Social Sciences database (SPSS V. 20). Student’s t-tests were performed to compare pre-test and post-test scores between groups. Chi-square and regression tests were used to determine the factors that contributed significantly to the variance in post-test scores.

## Results

The response rate was 91% for the intervention group (31 out of 34 students completed), and 78% for the control group (26 out of 33 students).

### Academic achievement

Statistical analysis of the mean scores for the control and intervention groups regarding the pre-test showed that the difference in the mean scores was not significant (Table 1) this indicate equivalence between the two groups regarding baseline knowledge of dental surgical forceps, which in turn support validity of this trial.

**Table 1:** t-Test: Means of control and intervention group

	Control group		Intervention group	
	Pre-test	Post-test	Pre-test	Post-test
Mean	5.87	5.42	5.94	8.34
Variance	8.73	7.61	5.35	6.17
Pearson correlation	0.71		0.13	
df	25		30	
P(T < =t) two-tail	0.31		0.00	

The T-test statistical analysis of the control group, showed that the difference of the mean scores regarding the pre and post-test was insignificant (*P* = 0.3). On the same format, statistical analysis of the mean scores of the intervention group regarding pre and post- test revealed that the difference is significant (*P* = 0.000) (Table 1).

**Fig. 3:**
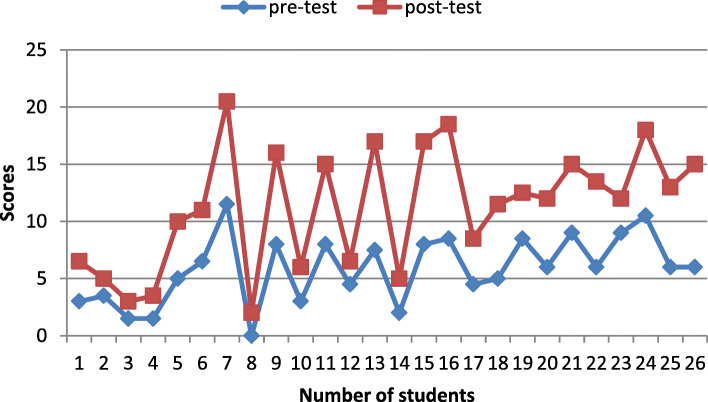
Stacked line chart showing comparison of pre-test and post-test scores of the control group. Primary horizontal axis title: Number of students Primary vertical axis title: scores. Chart legend: pre-test (blue line) post-test (red line)

**Fig. 4:**
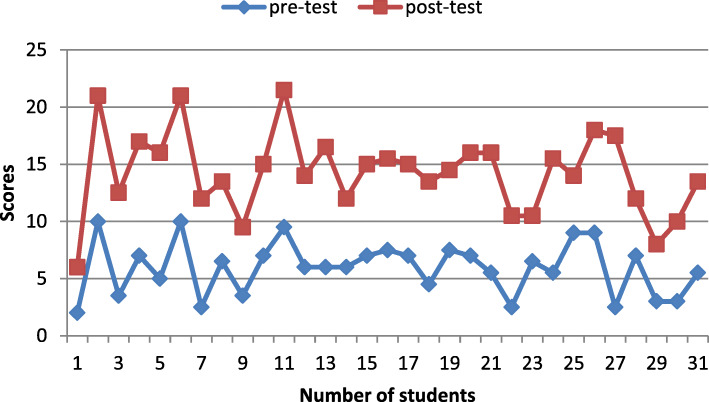
Stacked line chart showing comparison of pre-test and post-test scores of the intervention group. Primary horizontal axis title: Number of students Primary vertical axis title: scores. Chart legend: pre-test (blue line) post-test (red line)

Figures 3, and 4 shows the comparison results of the learning performance of the control and intervention group respectively (the students’ scores in the pre-test and post-test). The post-test results indicated that intervention group participants’ scores have obvious difference from their pre-test scores.

### Attitude section

Responses to each indicator of the questionnaire were given scores from 1 to 5 on a Likert Scale, ranging from “Strong agree = 5 to strong disagree = 1”. The mean score 3.0 was the bench mark, greater than 3.0 indicate positive perspective, below 3.0 indicate negative perspective (Table 2).

**Table 2:** The descriptive statistics for the students’ attitude regarding effectiveness of mobile learning

Item No.	Questionnaire indicator	Strongly agree N %	Agree N %	Uncer- tain N %	Disagree N %	Strongly disagree N %	Average	Stand- ard devia- tion
Q1	Mobile learning can be an effective method of learning as it can give immediate support.	17 54.8%	12 38.7%	2 6.5%	0 0	0 0	4.48	0.58
Q2	Mobile learning will bring new opportunities of learning	14 45.2%	15 48.4%	2 6.5%	0 0	0 0	4.41	0.64
Q3	Mobile learning will be more flexible method of learning as it can be done anytime, anywhere	19 61.3%	9 29%	3 9.7%	0 0	0 0	4.48	0.70
Q4	Mobile learning will improve communication between student and teacher.	9 29%	13 41.9%	6 19.4%	3 9.7%	0 0	3.85	0.99
	Mobile learning cannot be used for learning due to:							
Q5	expenses involved in Mobile learning	3 9.7%	10 32.3%	13 41.9%	5 16.1%	4 2.1	2.7	0.87
Q6	poor networking in the city	18 58.1%	11 35.5%	2 6.5%	0 0	0 0	1.48	0.64
Q7	unavailability of mobile phones with a larger number of students	2 6.5%	5 16.1%	14 45.2%	6 19.4%	4 12.9%	3.19	1.14

## Discussion

The result obtained from this research, indicate a marked difference in the students’ scores regarding their knowledge of dental surgical forceps, which clearly shows the effectiveness of mobile educational intervention (as a sort of m-learning) in increasing knowledge of the dental students. This result is consistent with previous studies on the effectiveness of m-learning intervention [10–12].

From an educational theoretical perspective, this can be explained by referring to Mayer’s cognitive theory of multimedia learning which assumes that people learn better from images when combined with words in an e-learning environment [13]. Mayer’s studies on using short multimedia tutorials also resulted in significant learning outcomes.

However, as with any learning process, we believe that information should be repeated continuously if knowledge is to be retained as there is deficient retention of knowledge after 30 days even when a teacher is available throughout the learning process [14] Thus, a probable advantage of mobile apps is that they make the information available all the time, repeatedly, accessed easily, and more cost-effective than printable text. This supported by the findings of a recent study regarding mobile learning in dentistry; in which the students defined the most favored features of m-learning as interactivity, easy accessibility, and repeatability [15].

The results of the students’ attitudes and perceptions on the effectiveness of mobile learning revealed that they generally positively reacted. The mean scores of the sample indicated that most of the participants (93.5%) responded positively to the first and second statements, which indicate that students believe in the new opportunities of learning that will be obtained by m- learning. The students also agreed in large part that m- learning is a more flexible method of learning as it can be done at any time, and anywhere. Concerning the fourth statement about (Mobile learning will improve communication between student and teacher), the responses varied from strongly agree to disagree, with a mean score of 3.85. This can be justified by the fact that the intervention used in this study was a “ready-made” App with no options for feedback or a discussion board.

The results of this part of the study are consistent with similar previous studies in which significant increases in attitude, and/or satisfaction scores after m-learning intervention were common findings [16–18]. Such general positive response and attitudes toward mobile learning can be explained by the fact that almost all students today own mobile phones and are very familiar with these devices [19].

Regarding the second part of the attitude section, the respondents cited concerns about the high cost involved in having and using mobile devices for m-learning. They also worry about the quality of the internet available to them. This result is consistent with other studies in different countries [20–22].

Although mobile educational intervention could improve the effectiveness in increasing knowledge for the dental students as it provides information access to students at anytime and anywhere, enhancing students’ satisfaction, encouraging self-centered learning, and facilitate interaction and collaboration between students and instructors through different communication activities [23] it cannot yet replace the traditional teaching method in dental education. Disadvantages may be summarized as follows; m-learning cannot provide practical hands-on lessons, smart-phone devices with its social media apps and games may cause a lot of distraction for students [24] also a lack of quality evidence-based, peer reviewed material represents one of the drawback [25].

### Limitations

Some limitations of this research should be considered, although the sample size was statically sufficient to compare the data of the two groups and support results, it was relatively small. A bigger sample size is mandatory to get more representive results, more valid and reliable conclusions. Second, the study conducted in a relatively short time (1 month), thus the retention of knowledge using this App on a long-term is uncertain. Longitudinal design is necessary to know the causal, and the retention effect of using mobile Apps.

## Conclusion

The results proved that smartphone educational application was effective tool of learning, regarding gaining and increasing knowledge.

The findings also reported positive attitudes and response of the dental students toward mobile learning.

## Supplementary Information

**Additional file 1.** Mobile application used in the study.

**Additional file 2.** Academic test.

**Additional file 3.** Attitude questionnaire.

## Data Availability

All data generated or analysed during this study are available from the corresponding author on reasonable request.

## Abbreviations

App: Application
ICT: Information and Communication Technology
M-learning: Mobile learning
