# Supplementary figures and images for: Effect of mobile learning on academic achievement and attitude of Sudanese dental students: a preliminary study
Source: BMC Med Educ. 2021 Feb 22;21:121. doi: 10.1186/s12909-021-02509-x (PMC7898729; doi:10.1186/s12909-021-02509-x)

## Additional File 1: Mobile application used in the study

Fig.1


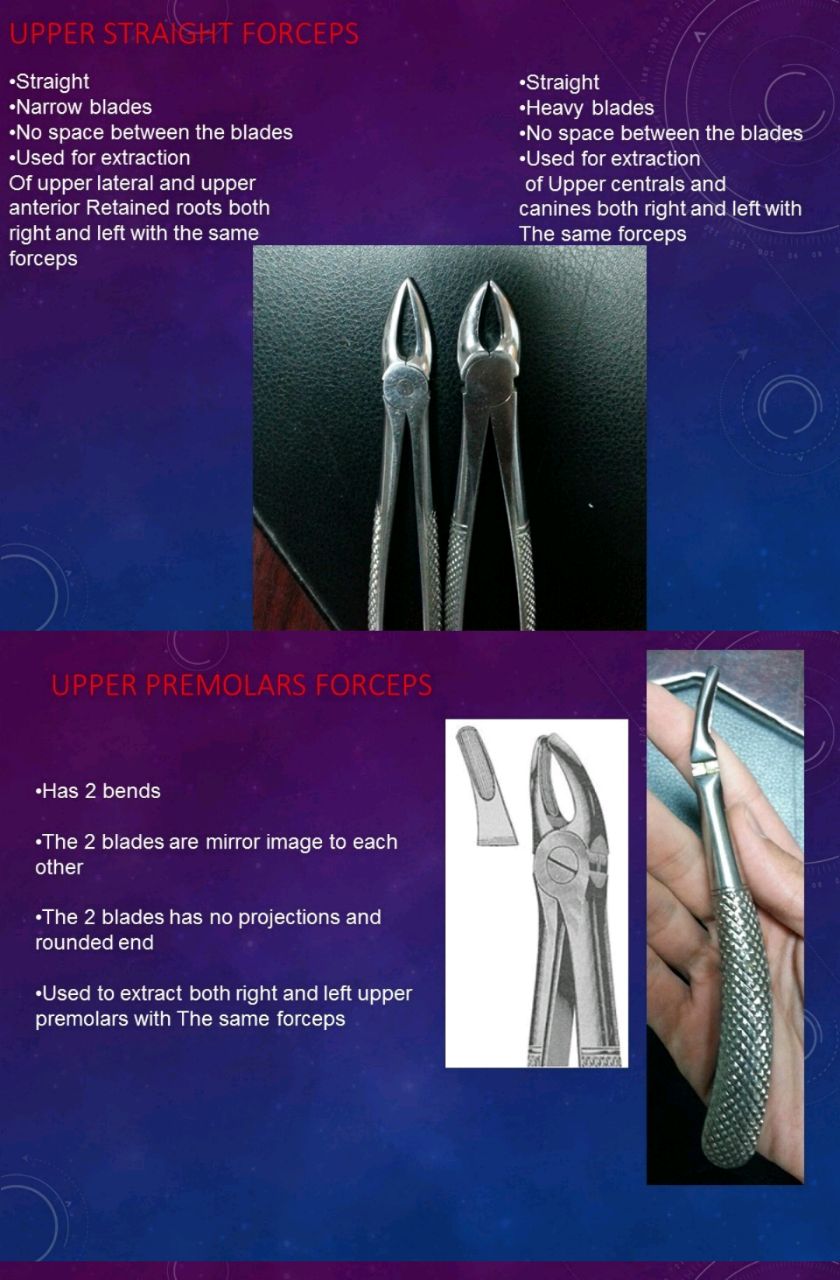


Fig.2


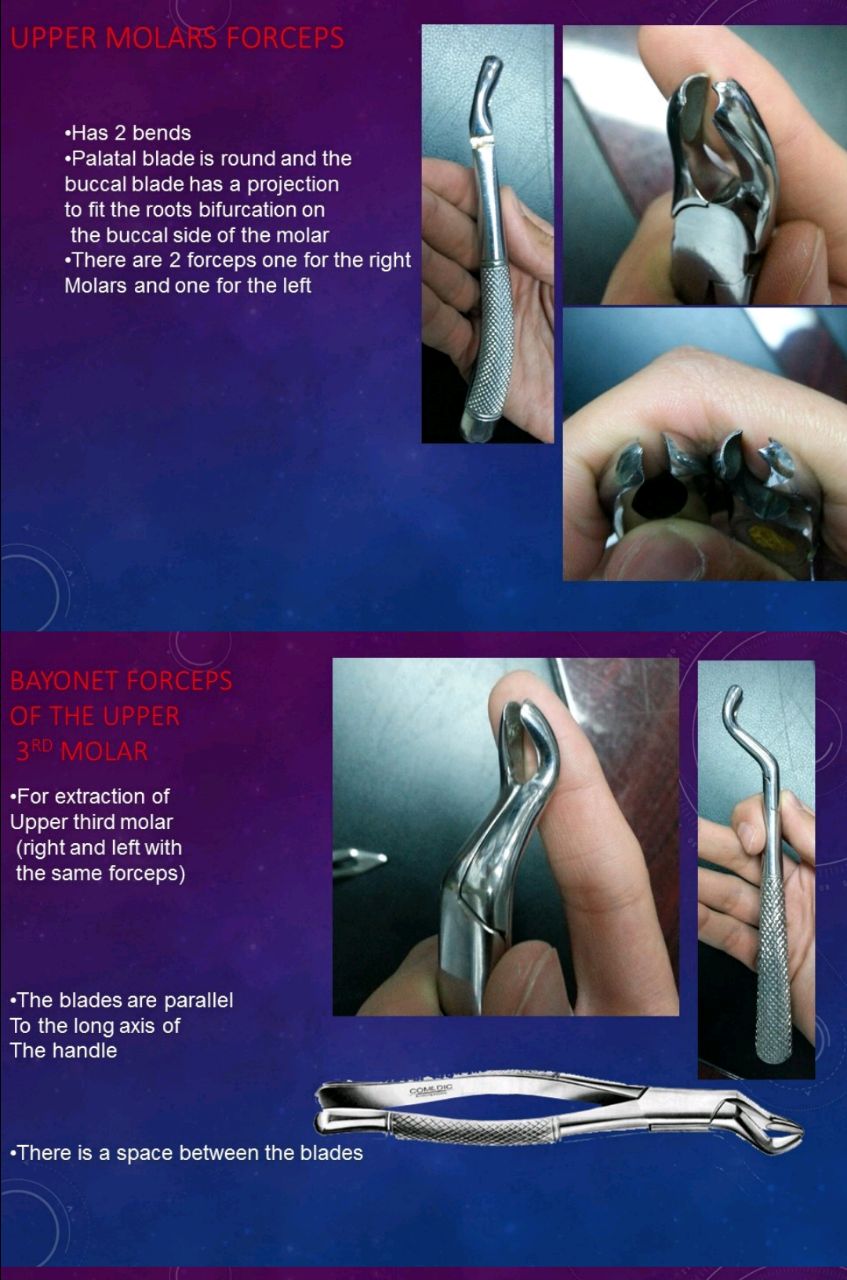


## Fig.3


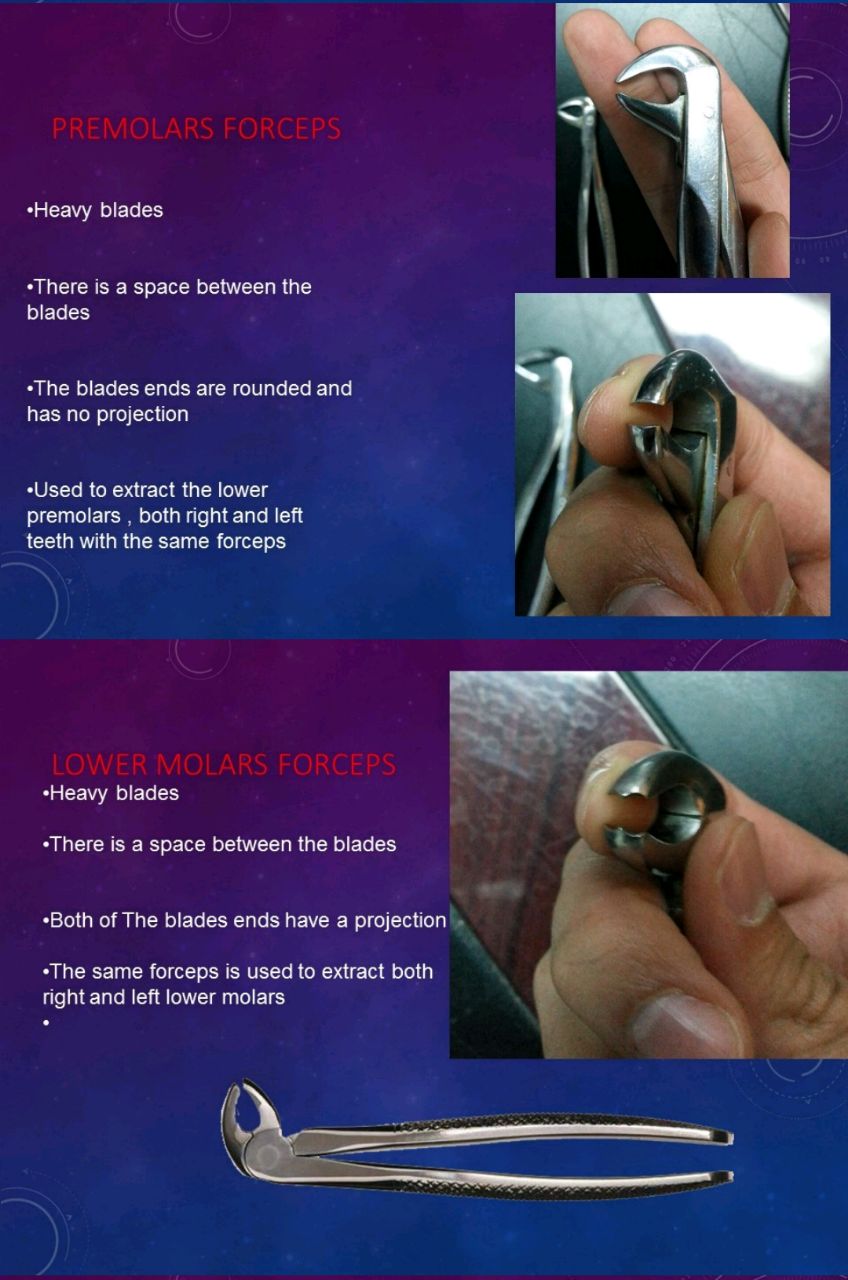


Fig.4


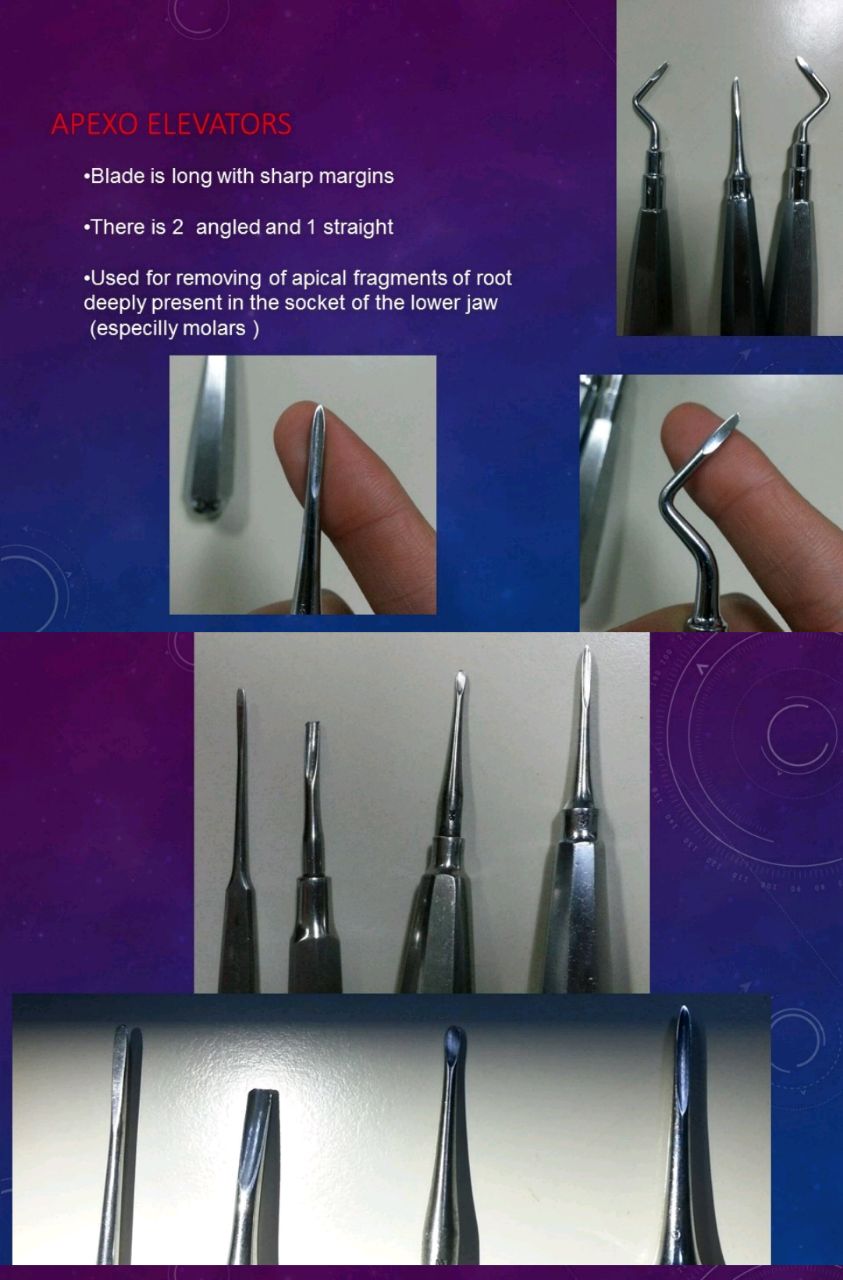


Fig.5

**
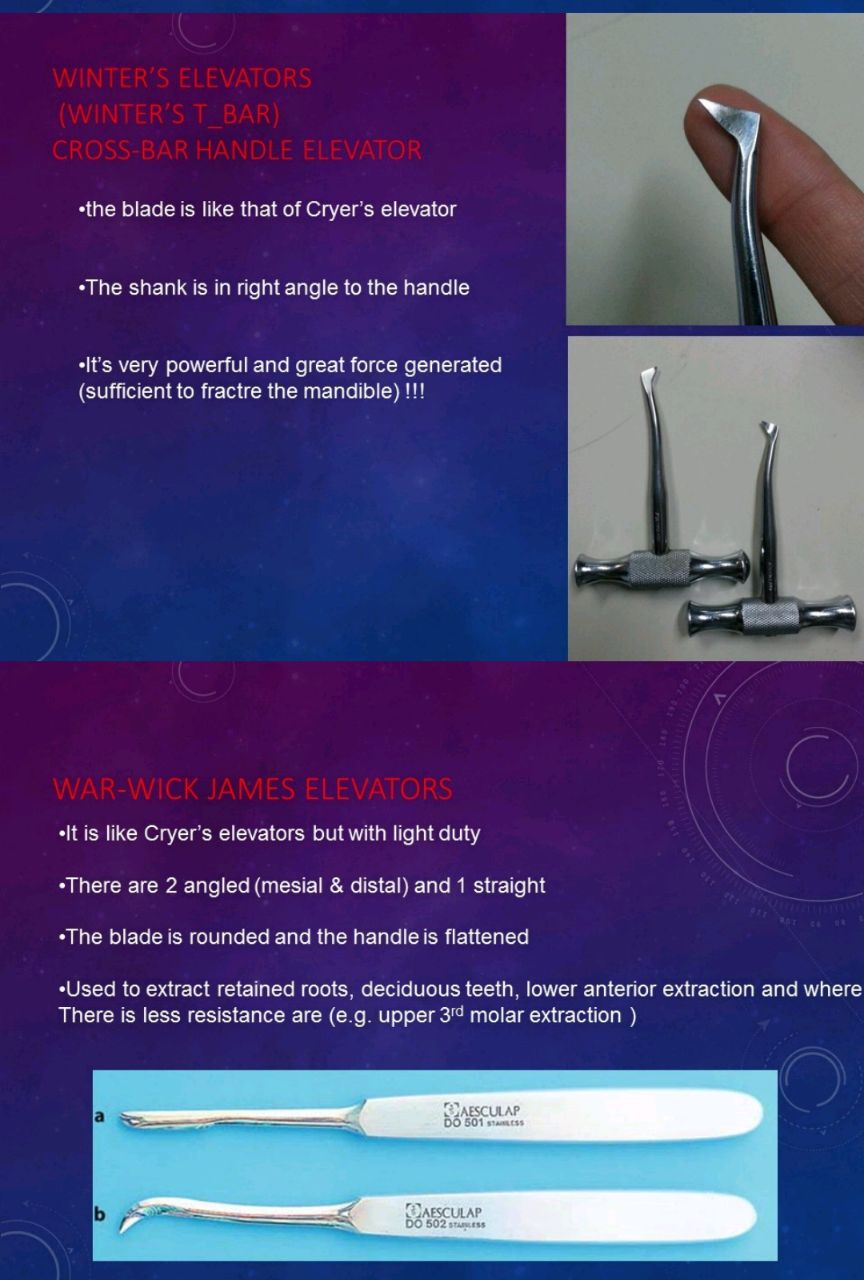
**

Fig.6

**
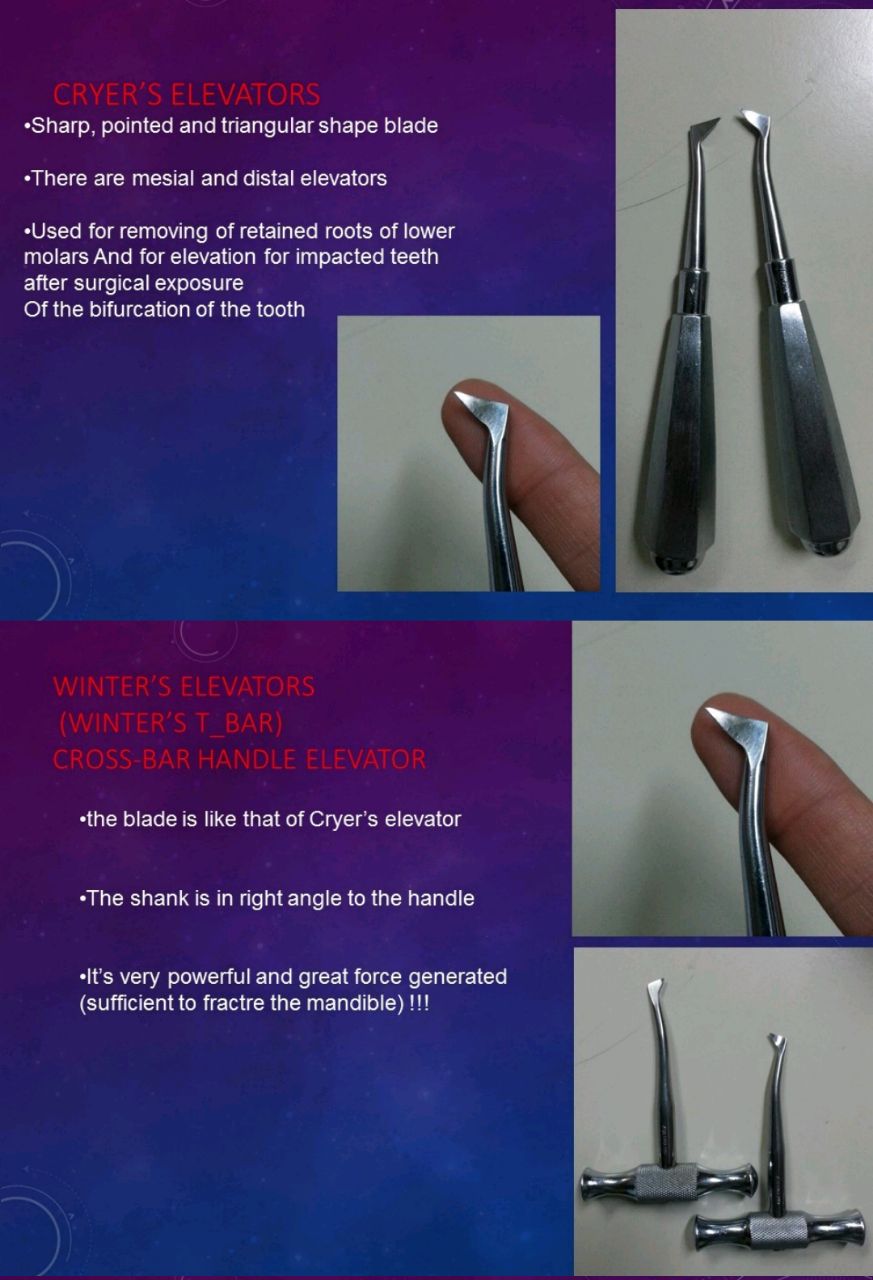
**

## 

Supplement: Supplementary file 1 — Additional file 1. Mobile application used in the study. [file 12909_2021_2509_MOESM1_ESM.docx]
