# Supplementary material for: Effect of mobile learning on academic achievement and attitude of Sudanese dental students: a preliminary study
Source: BMC Med Educ. 2021 Feb 22;21:121. doi: 10.1186/s12909-021-02509-x (PMC7898729; doi:10.1186/s12909-021-02509-x)
Supplement: Supplementary file 2 — Additional file 2. Academic test. [file 12909_2021_2509_MOESM2_ESM.docx]

**Additional File 2: academic test**

**Table 1: the pre-test**

|  | Instrument name | Used for |
| --- | --- | --- |
| 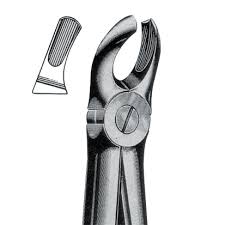 |  |  |
| 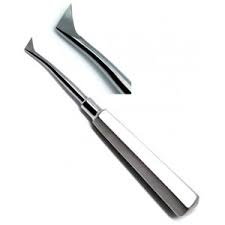 |  |  |
| 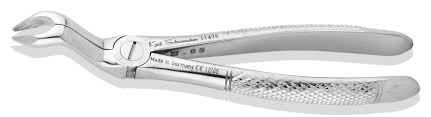 |  |  |
| 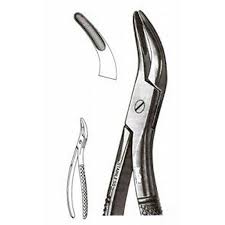 |  |  |
| 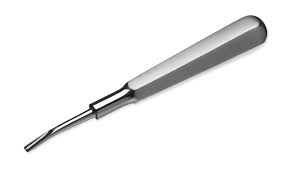 |  |  |
| 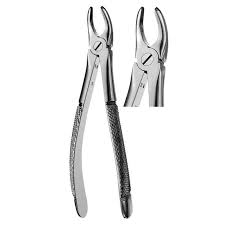 |  |  |
| 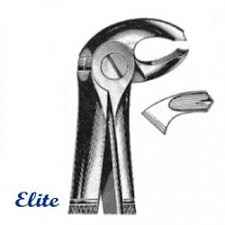 |  |  |
| 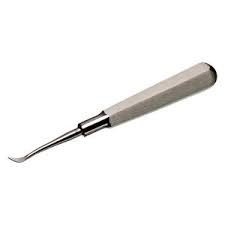 |  |  |
| 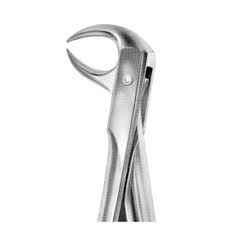 |  |  |
| 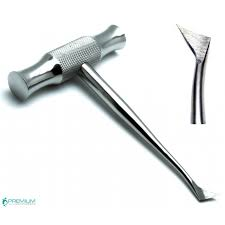 |  |  |

**Table 2: the post test**

|  | Instrument name | Used for |
| --- | --- | --- |
| 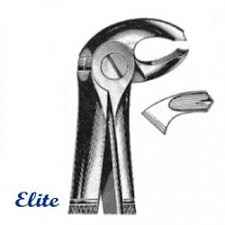 |  |  |
| 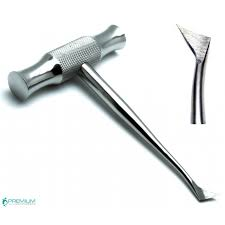 |  |  |
| 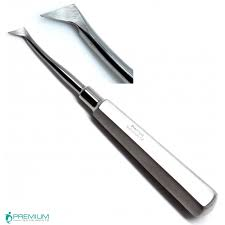 |  |  |
| 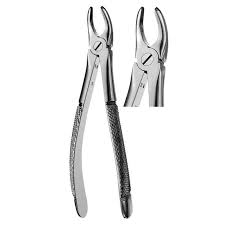 |  |  |
| 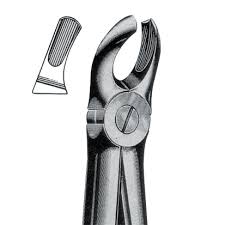 |  |  |
| 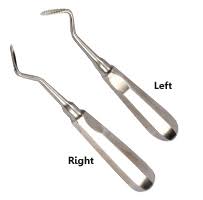 |  |  |
| 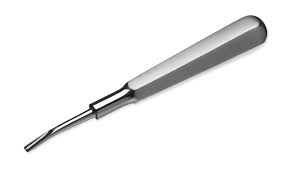 |  |  |
| 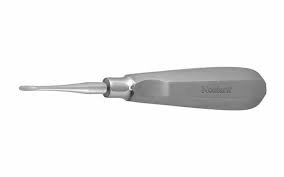 |  |  |
| 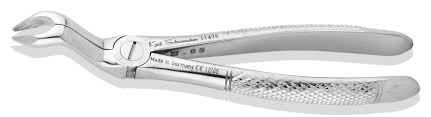 |  |  |
| 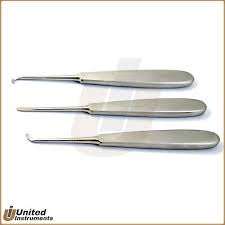 |  |  |
