# Supplementary material for: Effect of mobile learning on academic achievement and attitude of Sudanese dental students: a preliminary study
Source: BMC Med Educ. 2021 Feb 22;21:121. doi: 10.1186/s12909-021-02509-x (PMC7898729; doi:10.1186/s12909-021-02509-x)
Supplement: Supplementary file 3 — Additional file 3. Attitude questionnaire. [file 12909_2021_2509_MOESM3_ESM.docx]

Additional File 3: Attitude questionnaire

**Table 1**

|  | Strongly agree | Agree | Not sure | Disagree | Strongly disagree |
| --- | --- | --- | --- | --- | --- |
| 1. Mobile learning can be an effective method of learning as it can give immediate support |  |  |  |  |  |
| 1. Mobile learning will bring new opportunities of learning. |  |  |  |  |  |
| 1. Mobile learning will be more flexible method of learning as it can be done anytime, anywhere. |  |  |  |  |  |
| 1. Mobile learning will improve communication between student and teacher |  |  |  |  |  |
| Mobile learning cannot be used for learning due to: | | | | | |
| 1. Unavailability of mobile phones with a larger number of students. |  |  |  |  |  |
| 1. expenses involved in Mobile learning |  |  |  |  |  |
| 1. poor networking in the city |  |  |  |  |  |
